# Effect of Water Models on Transmembrane Self-Assembled Cyclic Peptide Nanotubes

**DOI:** 10.1021/acsnano.1c00155

**Published:** 2021-03-19

**Authors:** Martin Calvelo, Charlotte I. Lynch, Juan R. Granja, Mark S. P. Sansom, Rebeca Garcia-Fandiño

**Affiliations:** † Center for Research in Biological Chemistry and Molecular Materials (CIQUS), University of Santiago de Compostela, 15782 Santiago de Compostela, Spain; ‡ Department of Biochemistry, University of Oxford, South Parks Road, Oxford OX1 3QU, United Kingdom

**Keywords:** self-assembly, nanotubes, cyclic-peptide, water models, lipid bilayer, molecular dynamics

## Abstract

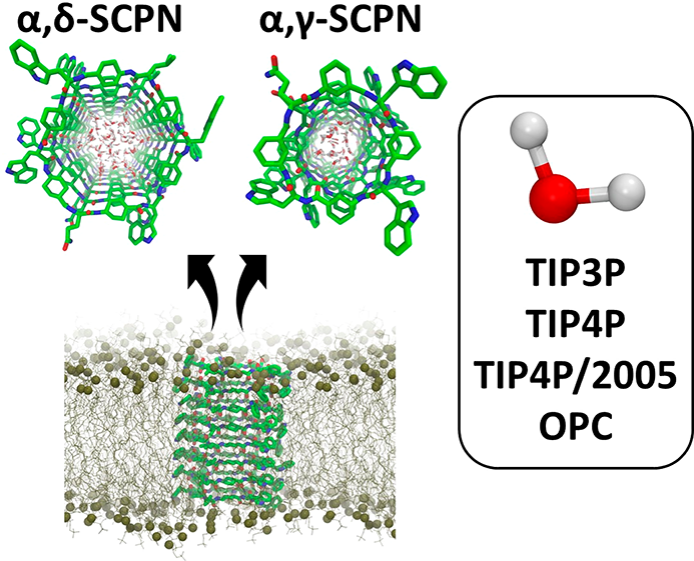

Self-assembling cyclic peptide nanotubes can form nanopores when they are inserted in lipid bilayers, acting as ion and/or water permeable channels. In order to improve the versatility of these systems, it is possible to specifically design cyclic peptides with a combination of natural and non-natural amino acids, enabling the control of the nature of the inner cavity of the channels. Here, the behavior of two types of self-assembling peptide motifs, alternating α-amino acids with γ- or δ-aminocycloalkanecarboxylic acids, is studied *via* molecular dynamics (MD) simulations. The behavior of water molecules in nanopores is expected to affect the properties of these channels and therefore merits detailed examination. A number of water models commonly used in MD simulations have been validated by how well they reproduce bulk water properties. However, it is less clear how these water models behave in the nanoconfined condition inside a channel. The behavior of four different water models—TIP3P, TIP4P, TIP4P/2005, and OPC—are evaluated in MD simulations of self-assembled cyclic peptide nanotubes of distinct composition and diameter. The dynamic behavior of the water molecules and ions in these designed artificial channels depends subtly on the water model used. TIP3P water molecules move faster than those of TIP4P, TIP4P/2005, and OPC. This demeanor is clearly observed in the filling of the nanotube, in water diffusion within the pore, and in the number and stability of hydrogen bonds of the peptides with water. It was also shown that the water model influences the simulated ion flux through the nanotubes, with TIP3P producing the greatest ion flux. Additionally, the two more recent models, TIP4P/2005 and OPC, which are known to reproduce the experimental self-diffusion coefficient of bulk water quite well, exhibit very similar results under the nanoconfined conditions studied here. Because none of these models have been parametrized specifically for waters confined in peptide nanotubes, this study provides a point of reference for further validation.

Water is one of the most studied molecules because of its importance in biological systems, as well as in other areas, including nanoscience, technology, and many industrial applications.^1^ The diverse properties of this solvent in the bulk solution, in nanoconfined environments, in solvation shells around other molecules, and at interfaces between media of different polarity have inspired the development of many water models for computational simulation studies.^2^ Despite the pioneering development of a realistic interaction potential for water by Bernal-Fowler in 1933,^3^ it was not until almost 40 years later that a computer calculation was carried out by Barker and Watts.^4^ Since then, more than a hundred different water models have been developed, in an effort to reproduce a number of experimental properties such as density, vaporization enthalpy, interfacial tension and molar heat capacity.^5−8^

Water molecules in the nanoconfined environment provided by natural or artificial membrane channels deserve special attention, because they are expected to behave significantly differently from those in bulk solution and at interfaces.^2,9^ Simplified models of nanotubes and nanopores have been extensively studied in terms of the behavior of nanoconfined water within their cavities, using both continuum fluid dynamics (CFD) theory and molecular dynamics (MD) simulations.^10−17^ Perhaps surprisingly, continuum models (modified by insights from atomistic MD simulations) have provided reasonable descriptions of the behavior of water in simple nanopores. Even with continuum models, the influence of the internal shape and hydrophobicity of the channels has been demonstrated.^18−20^ For example, studies carried out by Gravelle *et al.* with models of aquaporins concluded that the internal geometry of the channel plays a role, suggesting that structures which reduce the surface friction, as for example in an hourglass shape, favor water permeability when compared with cylindrical channels.^21,22^ However, it has been suggested that the accuracy of the continuum models depends on the hydrophobicity and size of the pore, such that water flow is underestimated for small hydrophobic pores (<1 nm).^2,23^ For small pores, the structural and dynamical properties of water are strongly influenced by interactions with the pore-lining interfaces, and thus, the detailed chemical properties of the pores become more important in determining water behavior. It is likely that for the design of nanopores and in order to understand complex biological nanopores, accurate atomistic simulations of water properties are required. In this context, MD simulations emerge as a good alternative for studying pores with small radii (<1 nm).^2^

MD studies have shown that the behavior of different biological structures can depend on the water model employed. For example, Anandakrishnan *et al.* have recently shown the importance of the water model in the calculations of protein folding landscapes,^24^ and similar conclusions have been obtained for RNA.^25^ Host–guest binding energy differences for supramolecular complexes based on cyclodextrins have proved to depend significantly on the water model.^26^ Some computational studies with cylindrical systems embedded in lipid bilayers, acting as transmembrane channels, also suggest differences depending on the selected water model.^27−30^ For example, the extensive work of Kassinos *et al.* on carbon nanotubes (CNTs) shows differences in water density, self-diffusivity, and even in the stability of CNTs.^27−29^ Liu *et al.* investigated pressure driven flow rates of water through a (6,6) CNT for the TIP3P, SPC/E, and TIP4P/2005 water models, finding a high dependence of the flow rates on the water model, with TIP3P showing the fastest flow and TIP4P/2005 the slowest.^31^ In contrast to larger [(8,8) and (9,9)] CNTs considered in earlier works, the different flow rates cannot be attributed to different model-dependent water structures within the nanotubes but to differing bulk mobilities of the water models affecting the rate of entry into the nanotube.^32^ A recent extensive survey of simulations of water behavior in nanopores and channels also reveals a number of cases where the choice of water model influences the behavior of water in a nanopore environment, especially in hydrophobic gating of ion channels.^2,30^ Thus, the selection of the water model for such simulations deserves particular scrutiny. This is because water models are typically parametrized and evaluated by how well they can reproduce the properties of bulk water, and the extent of transferability of such water models to nanoconfined water remains unclear. Furthermore, experimental data are not available for these systems. Therefore, to date, the best practice for MD simulations involving water in confined systems is to compare results obtained with different water models.

Recent studies have shown that the presence of hydrophobic regions in different membrane channel proteins can play an important role in controlling the transport of ions, water, and other solutes.^33−37^ These regions can be wetted upon application of an electric field and then dewetted after the field removal, facilitating the translocation of small charged species, and also of single-stranded DNA molecules.^34^ Thus, the study of artificial hydrophobic nanopores that mimic the behavior of the natural channels has attracted growing interest in using them as common platforms for technological applications, including those where nanopores are used as sensors, such as in DNA sequencing devices.^32^ Self-assembled cyclic peptide nanotubes (SCPNs) could prove interesting for such a task, given their cylindrical structures with a partially hydrophobic inner cavity.^38−41^ In addition, their synthetic simplicity makes them readily available for exploring their properties and would facilitate its commercialization. As a result of their biocompatibility, the tuneability of their diameters, and the range of possibilities for functionalizing their inner cavities, SCPNs have emerged as potential candidates for functional transmembrane channels.^38,39,49−52,40,42−48^ The SCPNs were originally synthesized by Ghadiri *et al.* in 1993 using a combination of cyclic peptides (CPs) formed by *D* and *L* α-amino acids (Aas).^53^ This chiral alternation in the sequence provides pleated structures that self-assemble by β-type hydrogen bonds (H-bonds) between backbone atoms of adjacent rings.^54^ In such structures the donor and acceptor groups of each amide residue (NH and C=O) point in the same direction perpendicular to the plane of the ring (Figure 1A). Since then, CPs using different kinds of amino acids have been developed, obtaining structures with tuned properties. For example, cyclic γ-amino acids (*cis*-γ-aminocycloalkanecarboxylic acids, γ-Acas) alternated with α-amino acids (**α,γ-CPs**) can also form SCPNs following the same principles described above (Figure 1A).^42,55−59^ In these structures, the β-carbon of the cycloalkane is directed toward the lumen of the cylinder, influencing the internal properties of the nanotube and allowing further chemical modification. Other types of nanotubes with hydrophobic cavities can be obtained using the *trans*-4-aminocyclohexanecarboxylic acid (δ-Ach) as a building block (Figure 1A).^60^ The resulting nanotubes formed by stacking of α,δ-CPs have a hydrophobic internal cavity because two of the methylenes of each cyclohexyl moiety are oriented toward the inner cavity. While the interaction and transport of water by SCPNs composed by the classical *D*,*L*-α-CPs have already been studied, no comparable characterizations have been performed in systems using nonstandard amino acids that might lead to structures with other properties.^61−65^ A good understanding of the behavior of these SCPNs provides useful information for the design and optimization of artificial nanopores, as well as for the further development of water models and the understanding of their interactions with biological molecules. In addition, the present study will also shed light on the structural stability and behavior of this class of peptide nanotubes: α,δ-SCPNs.

**Figure 1:**
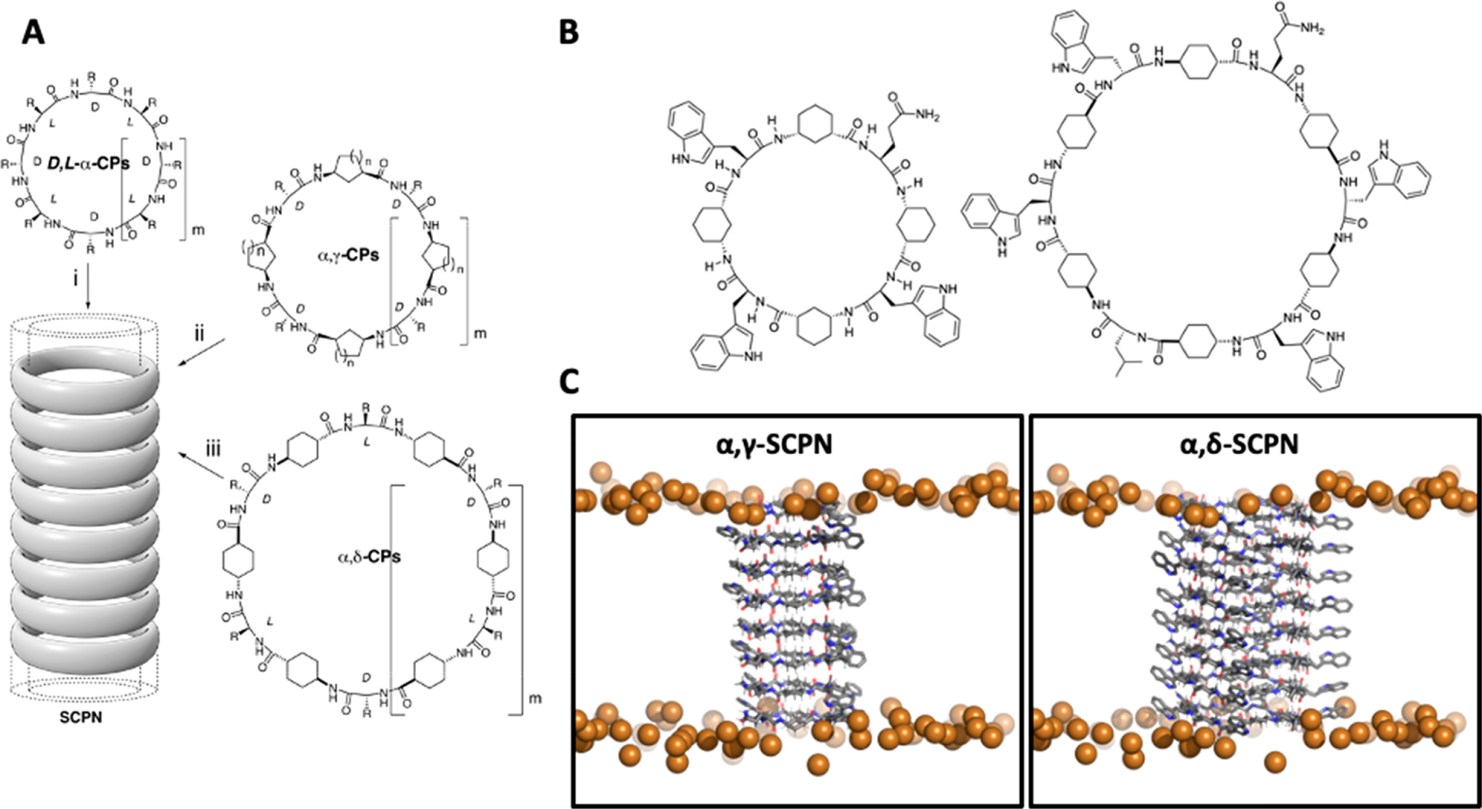
(A) Peptide nanotube models formed by the stacking of cyclic peptides of different types: (i) *D*,*L*-α-CP, (ii) CPs containing γ-Acas (α,γ-CPs) and (iii) CPs containing δ-Acas (α,δ-CPs). (B) CP sequences studied in this work. Left: α,γ-CP; Right: α,δ-CP. (C) Initial structures for the MD simulations of α,γ-SCPN (left) and α,δ-SCPN (right). For clarity, only the SCPN and the phosphorus atoms of the lipid molecules forming the bilayer (brown spheres) are represented.

In this study we describe in detail the structural stability of two kinds of SCPNs inserted into a phospholipid bilayer, α,γ-SCPNs and α,δ-SCPNs (Figure 1B,C), alongside simulation of their interactions with four water models, two of which (TIP3P and TIP4P) have been used in many studies of biomolecules and nanopores, as well as two more recently developed water models (TIP4P/2005 and OPC).^66−69^ Whereas the α,γ-SCPN is composed of eight residue-long CPs (four α-Aa and four γ-aminocyclohexanecarboxylic acid), the α,δ-CPs of the latter SCPN contain 12 amino acid residues (6 α-Aa and 6 δ-aminocyclohexanecarboxylic acid). The larger diameter of the second nanopore allows for the encapsulation of systems as large as C_60_ moieties.^60^ Taken together these studies provide a systematic comparison of the effect of different water models in MD simulations of nanoconfined water in two different sizes of self-assembling cyclic peptide nanotubes.

## Results and Discussion

### Models

In order for the nanotube to be long enough to traverse the membrane, and following previous studies, an α,γ-SCPN structure composed of eight CPs was built, with four α-Aa and four γ-aminocyclohexanecarboxylic acid residues (*c*-[*L*-Gln-*D*-γ-Ach-(*L*-Trp-*D*-γ-Ach-)_3_]) in each ring.^61,70−73^ The α,δ-SCPN structure was also composed of eight CPs, but in this case each ring was made up of six α-Aa and six δ-aminocyclohexanecarboxylic acid residues (*c*-[*L*-Gln-δ-Ach-(*L*-Trp-δ-Ach-)_2_*L*-Leu-δ-Ach-(*L*-Trp-δ-Ach-)_2_]). The antiparallel and parallel configurations were chosen for α,γ-SCPN and α,δ-SCPN, respectively, based on preliminary studies.^74^ Because of the different number of amino acids, the initial minimum internal radii for the α,γ- and α,δ-SCPNs were 0.34 and 0.53 nm, respectively (Figure 1SIA). As mentioned above, the size of these nanotubes justifies the use of atomistic MD simulations as the best methodology for their study. Additionally, the different number of methylene groups oriented inward leads to a more hydrophobic cavity for the α,δ-SCPNs (Figure 1SIB).

The behavior of four water models (TIP3P, TIP4P, TIP4P/2005, and OPC) inside the nanopore lumen of these SCPNs embedded in a POPC (2-oleoyl-1-palmitoyl-*sn*-glycero-3-phosphocholine) membrane was studied (Figure 1B,C).^66−68^ TIP3P is a 3-point model; that is, it is composed of three particles: two positive point charges on the hydrogen sites and one negative point charge on the oxygen site. Each site also has a Lennard-Jones potential to describe the nondirectional interactions of the atoms. By contrast, TIP4P, and the more recently developed TIP4P/2005 and OPC models, are 4-point models. In this case the Lennard-Jones potential remains on the oxygen site, but the negative charge is displaced from the oxygen site toward the hydrogen sites. The geometries, Lennard-Jones parameters, and charges also differ between the models.^2,8,75^

All the simulations were performed in the presence of NaCl 0.15 M. For each system, five 50 ns MD trajectories were generated (giving a total of 250 ns per system).

### Structural Stability of the Nanopores

**Figure 2:**
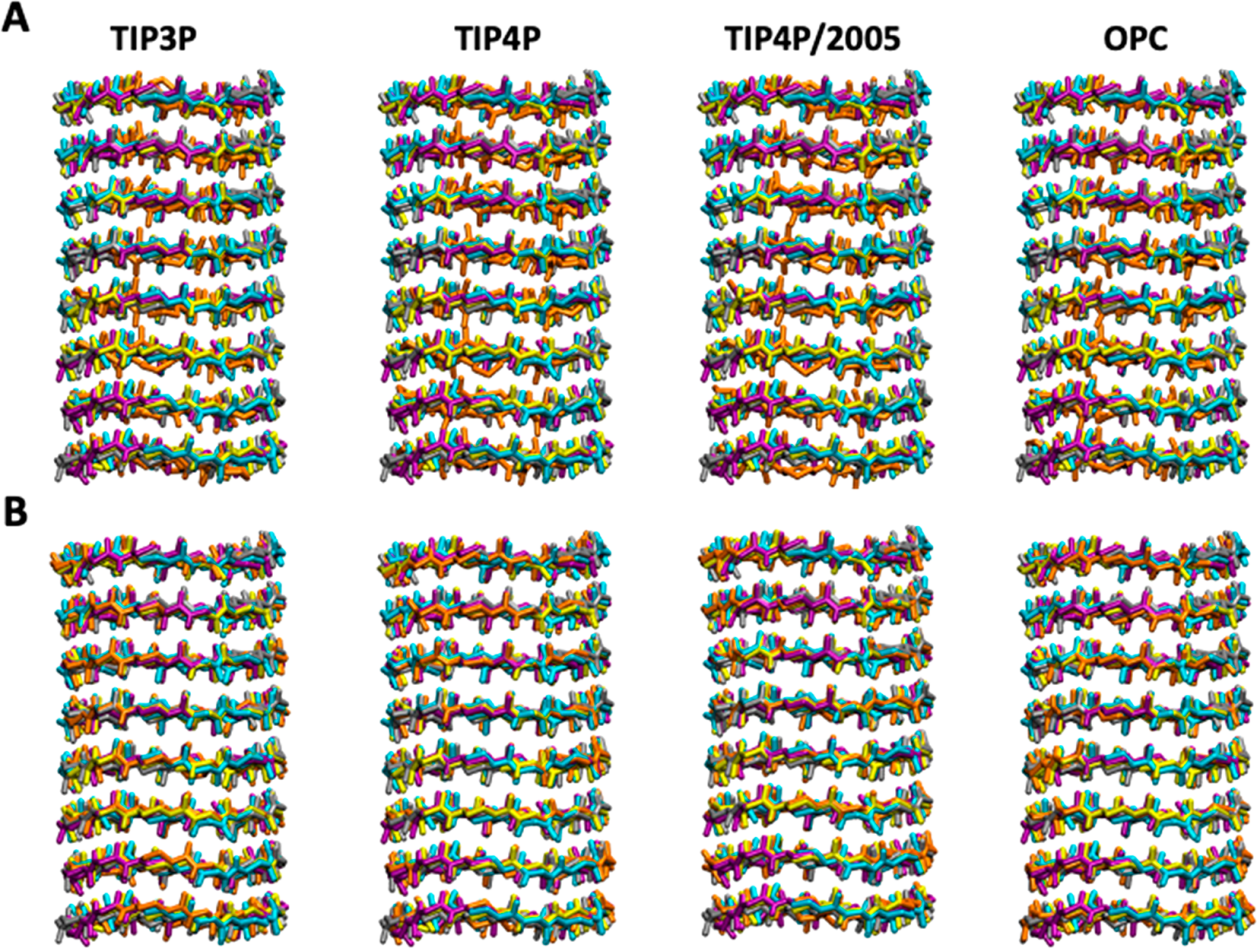
Snapshots of the backbone of a simulated α,γ-SCPN (A) and α,δ-SCPN (B) after 50 ns of simulation. The final structure of each replica is represented in a different color (5 replicas per water model, 5 colors).

**Figure 3:**
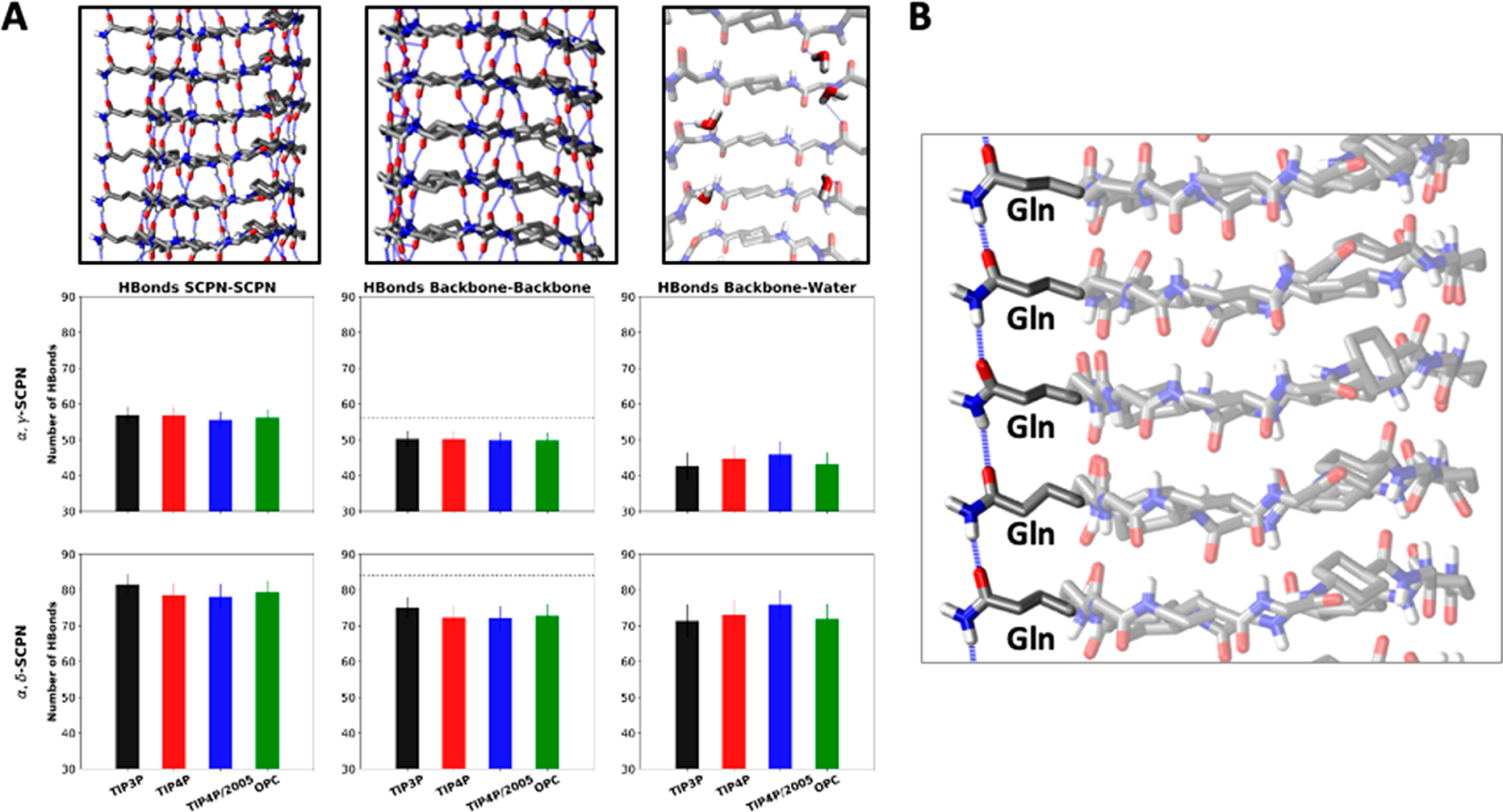
(A) Number of H-bonds between the different parts of the system (among the different CPs, as a whole or just considering the backbone, and between the CPs and the water), averaged over the last 40 ns of the simulation, and over all replicas. Standard deviations are also shown as error bars. A detail of each type of interaction is shown in the panels at the top. The maximum number of H-bonds that can be formed among the backbone of all CPs is displayed with a dashed line. (B) Detail of the Gln-Gln H-bonds, shown as broken blue lines.

Previous studies with α,γ-SCPNs composed of *c*-[(l-Trp-D-γ-Ach-)_4_] have shown that they are stable in a lipid bilayer environment, forming nanopores.^71,72,76,77^ α,δ-SCPNs have not yet been studied, so no evidence on their structural stability exist. Both the bilayer and nanotubes were stable in the 40 MD trajectories (two different nanotubes, α,γ-SCPNs and α,δ-SCPNs, each simulated with the four water models, with five replicas for each simulation) (Figure 2). This confirms that α,γ-SCPNs are stable, as previously observed, and indicates that α,δ-SCPNs can function as transmembrane channels (Figure 2B). The tubular shape is well-preserved during the simulation time, with the average root mean square deviation (RMSD) values for the α,γ-SCPN being slightly smaller than the ones for the α,δ-SCPN (Figure 2SI). No significant differences in pore model stability are found between the trajectories using different water models. The RMSD analysis by CP position in the nanotube also demonstrated the stability of the nanotube, although the CPs at either end of the nanotubes exhibit a slightly larger movement than the others because of reduced packing interactions (Figure 3SI).

The number of H-bonds is directly related to the stability of the nanotube. In the case of α,γ-SCPN, the number of H-bonds between the backbone of the CPs (49 ± 2, Figure 3A) is close to the maximum possible number of H-bonds that can be ideally formed (56 = 8 per CP × 7 pairs of CPs), whereas for α,δ-SCPN the number of H-bonds is 72 ± 3 (compared to the maximum of 84 = 12 per CP × 7 pairs of CPs) (Figure 3A). These results show that the sacrifice of almost 15% of the interbackbone H-bonds, probably because of competition with the water, is not enough to disrupt the tubular structure of the channel. Furthermore, some extra H-bonds (around 6–7) are formed between the different CPs apart from those corresponding to the structural backbone network. These interactions correspond to the Gln-Gln side-chain H-bonds that are present in both nanotubes (see atomistic detail in Figure 3B). Regarding the comparison between the water models employed, no significant differences are found apart from a slightly increased number of H-bonds in the nanotube network for the α,δ-SCPN in TIP3P (Figure 3A) and an increase in the number of H-bonds between the nanotube and water in both nanotubes when using TIP4P/2005 (Figure 3A).

**Figure 4:**
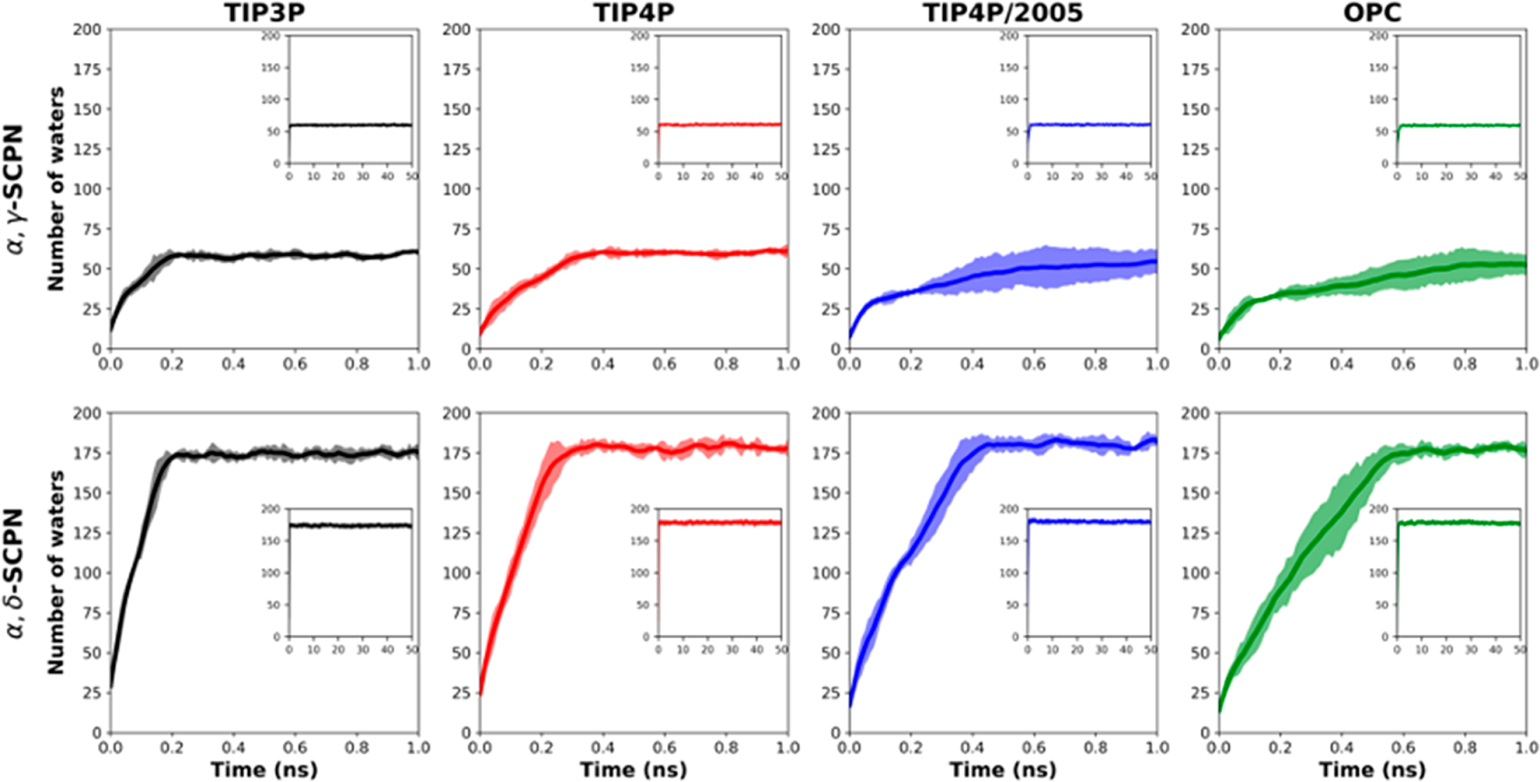
Number of waters entering α,γ-SCPNs (top) and α,δ-SCPNs (bottom) during the first 1 ns of each MD simulation. Each color corresponds to a different water model averaged over the five replicas. Standard deviations are shown in a paler color. The number of waters inside the nanotube over the total 50 ns of simulation is presented in the small insets.

The stability of the pore radii is a crucial issue from the point of view of the study of water and ion channels. The average values of the minimum radius averaged during the last 40 ns of the five replicas shows a quite constant inner size, with values very similar to the initial radius (Figure 4SIA). This stability suggests that these pores remain in an open state throughout the entirety of their trajectories, which corresponds to relatively long-lived channel openings observed experimentally.^71,78^ As has been previously observed for the α,γ-SCPNs, the effective radius is situated in the plane of the CP, whereas the maximum radius is located in the region between the two planes of the rings (Figure 4SIB).^71,72^ The smallest radii, corresponding to the plane of the CPs, alternate from smaller to bigger values from one CP to the next along the nanotube. The same trend is found for the α,δ-SCPN (Figure 4SIB). These differences in the size of the inner cavity could potentially lead to different transport behaviors and water confinement patterns (*vide infra*).

### Water Filling of the Channels

Because of the unequal internal radii and hydrophobic character of both nanotubes, differences in the entrance of molecules inside of the channels can be anticipated (Figures 1 and 2SI). The study of the filling of both channels reveals a faster process for TIP3P than for the other three water models, with the channel being completely full within the first 0.2 ns (Figure 4). Additionally, and especially in the α,δ-SCPNs, it is possible to observe that OPC waters need more time for a complete filling of the nanotube, suggesting that the diffusion of this water model within the pore may be slower than the others (Figure 4). TIP4P and TIP4P/2005 also show unequal behaviors, with TIP4P being more similar to TIP3P and TIP4P/2005 to OPC.

### Water Molecules Inside the Nanotubes

**Figure 5:**
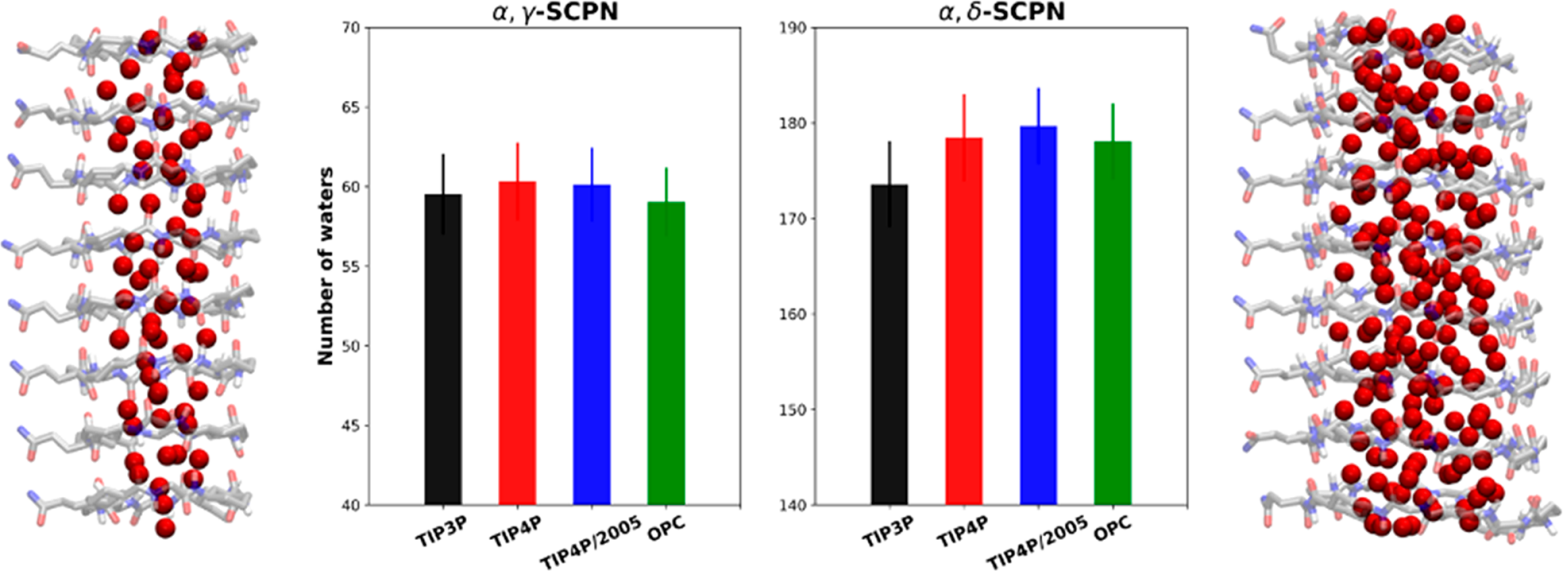
Number of waters inside α,γ-SCPNs and α,δ-SCPNs, averaged over the last 40 ns of the simulation, and over all replicas. Each color corresponds to a different water model. A picture of each channel with water inside (red spheres correspond to the water oxygen atoms) is shown for each SCPN. Standard deviations are also shown as error bars.

Because of the smaller radius of α,γ-SCPN, the average number of waters inside is significantly lower (more than 100 molecules less) compared to α,δ-SCPN (Figure 5). For both channels, simulations with TIP4P and TIP4P/2005 lead to the inclusion of more water molecules than with the other models. This may explain the higher number of H-bonds between the nanotubes and the water molecules (Figure 3A). Surprisingly, in the wider nanotube (α,δ-SCPN), a smaller number of TIP3P water molecules is found inside, despite being the water model which filled the nanotube fastest. This behavior is not observed for the α,γ-SCPN, where the differences between all water models are not significant. These observations suggest that there is no correlation between filling rate and the number of waters within the channel, suggesting that the number of waters inside is an equilibrium property and does not simply reflect differences in filling rates of the pores.

**Figure 6:**
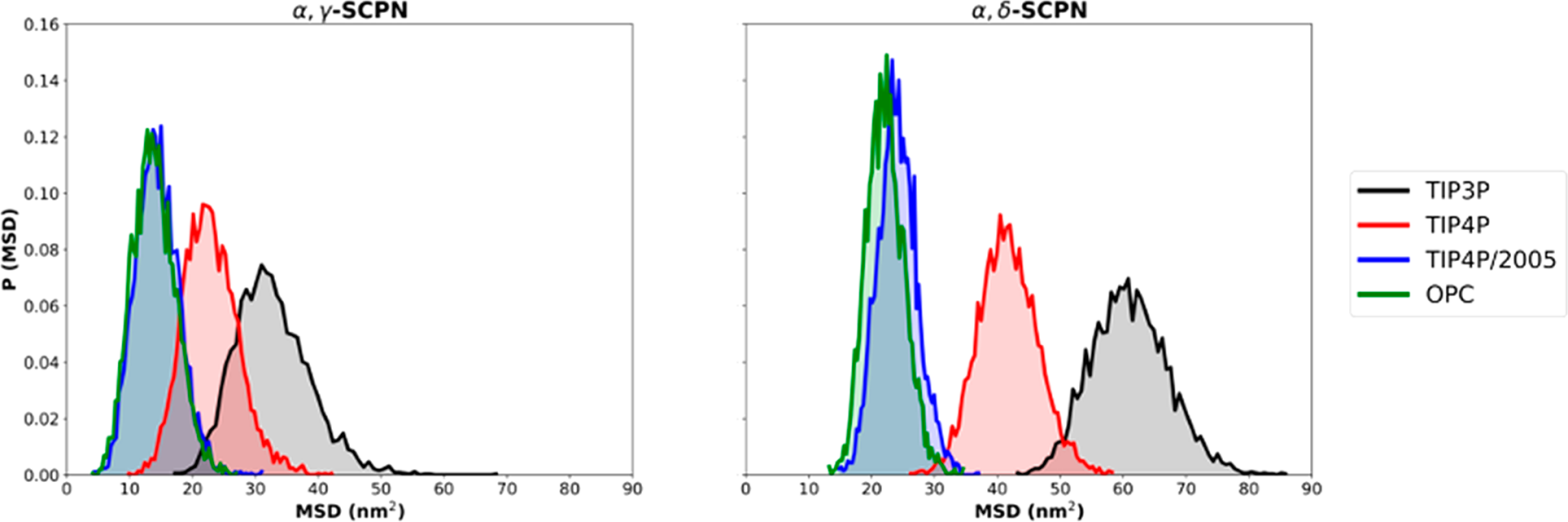
Probability distribution of the MSD of waters inside α,γ-SCPNs and α,δ-SCPNs during windows of 100 ps along the last 40 ns of all replicas for the different water models studied. Each water model is displayed with a different color (TIP3P in black, TIP4P in red, TIP4P/2005 in blue, and OPC green).

The velocity of water molecules inside both nanotubes was analyzed by calculating the mean square deviation (MSD) of all the encapsulated molecules during a time window of 100 ps (Figure 6). These results show that the TIP3P water molecules move more than the others in the same time interval, and TIP3P is therefore the fastest, followed by TIP4P. On the other hand, TIP4P/2005 and OPC are significantly slower than the other models. The velocity exhibited by these two parametrizations is very similar in both nanotubes. This trend reflects the self-diffusion in bulk water of the different models (*D* = 5.5, 3.2, 2.1, and 2.4 × 10^9^ m^2^ s^–1^ for TIP3P, TIP4P, TIP4P/2005, and OPC, respectively, compared to 2.3 × 10^9^ m^2^ s^–1^ experimentally).^66−68,79,80^ Similar trends were also observed for studies of pressure-induced flow through CNTs with radii between ∼0.4 and ∼0.9 nm, suggesting a nearly 3-fold difference between, for example, TIP3P and TIP4P/2005.^81^ Thus, TIP4P/2005 and OPC, which have very similar self-diffusion coefficients in bulk water (in good agreement with the experimental value), keep this similarity when the water is confined in a SPCN. TIP3P, which is known to diffuse too quickly in the bulk compared with experimental values, is also revealed as the fastest in this confined environment. Finally, an intermediate behavior is exhibited by TIP4P. Larger differences are found for α,δ-SCPN than α,γ-SCPN, with it being possible to observe a small overlap between the different water models in the narrower nanotube, especially for TIP3P and TIP4P. Furthermore, the same conclusion is obtained when longer time windows (200 and 500 ps) are defined (Figure 5SI). Additionally, and because of the larger inner radii, the water MSDs are greater for α,δ-SCPN (Figure 6).

**Figure 7:**
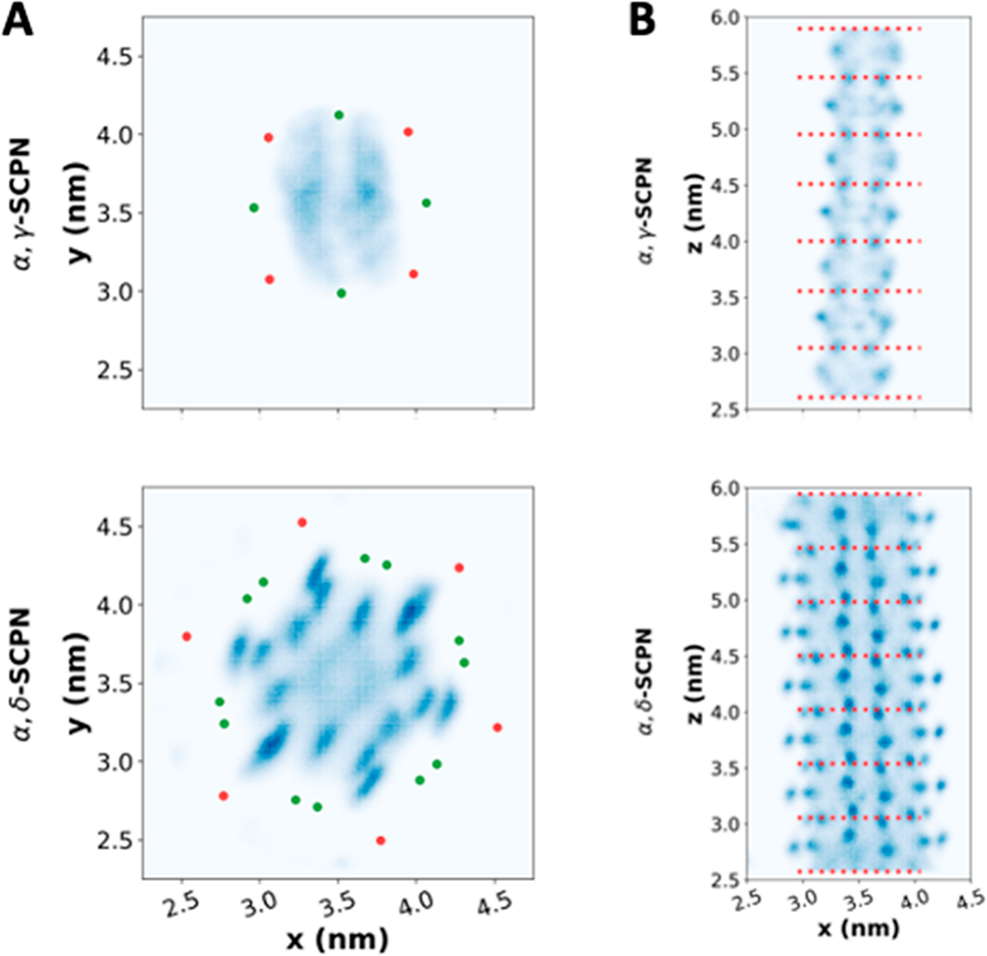
(A) *XY*-density profile of the water molecules inside both nanotubes. The averaged positions C_α_ of the α-amino acids and the C of the inward methylenes of the non-natural residues (one for the γ-Ach and two for the δ-residue) are highlighted in red and green, respectively. Only one water model (TIP4P/2005) is presented. The rest of them are available in Figure 8SIA. (B) *Z*-density profile of the water molecules inside both nanotubes. The averaged *Z*-coordinates of the α-carbons of each CP are highlighted with a red dashed line. Only one water model (TIP4P/2005) is presented. The rest of them are available in Figure 8SIB.

Differences between water models also appear when the water–peptide interaction strength is considered. Despite the similarity in the number of H-bonds between the nanotube and water (Figure 3), the lifetime of those bonds is longer for TIP4P/2005 and OPC, which could slow down their movement inside the SCPN (Figure 6SI). The H-bonds with the TIP3P waters are the most short-lived, followed by TIP4P. These results suggest a relation between the velocity of the water molecules inside the SCPN and the duration of the interaction with the peptide. Moreover, the obtained trend is also reflected in the survival probability of the waters inside the SCPN, in which TIP4P/2005 and OPC water are more likely to spend more time inside the nanotube than are TIP3P and TIP4P (Figure 7SI).

**Figure 8:**
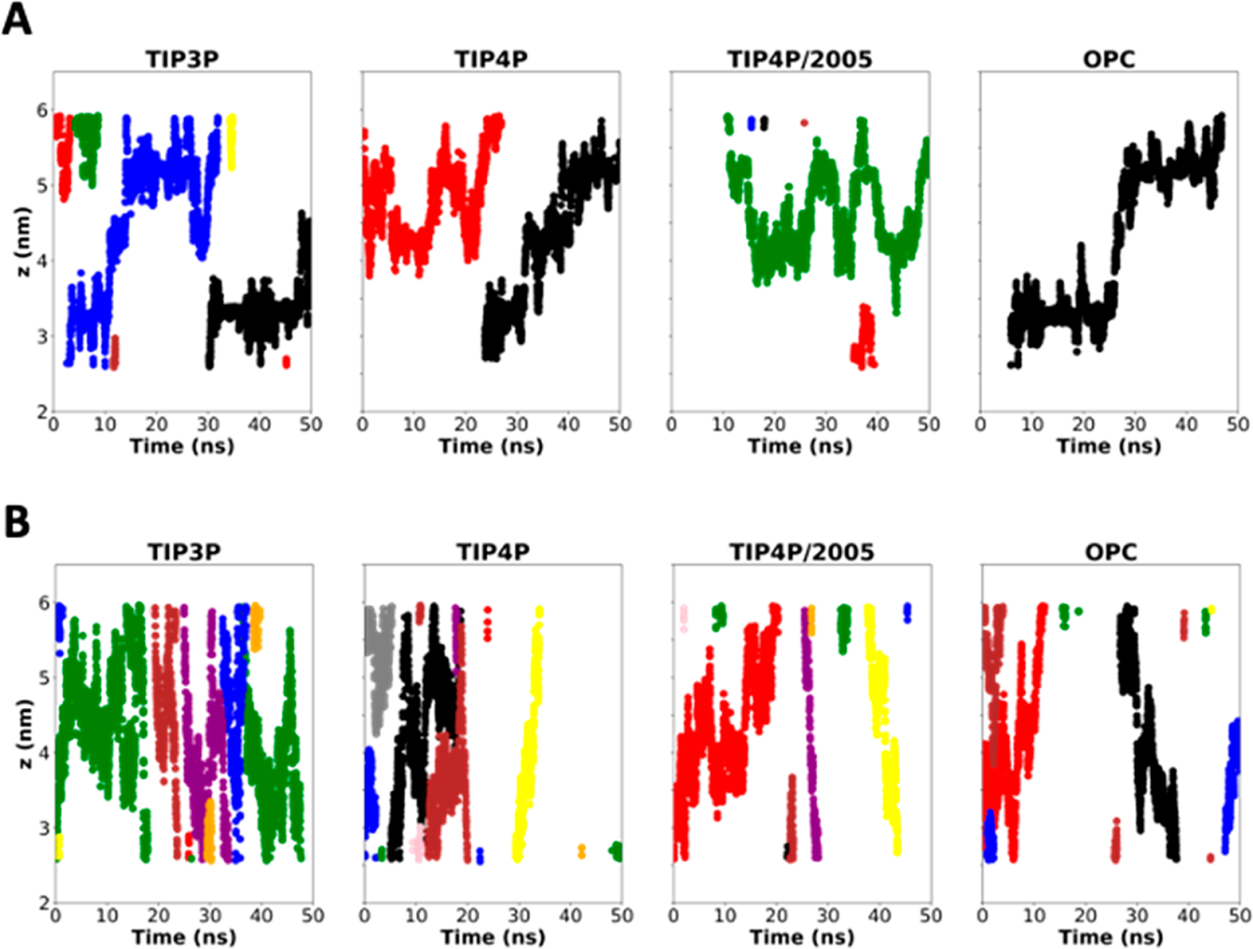
*Z*-coordinate for each of the cations inside the α,γ-SCPN (A) and α,δ-SCPN (B) along the 50 ns trajectory. The nanotube *z*-region is taken to be between ∼2 and 6 nm. Only one replica is presented. The rest are available in Figure 10SI. Each color corresponds to a different ion.

The different inner radii of the nanotubes also lead to an unequal pattern of water distribution inside the pores. The density of the encapsulated waters projected onto the membrane plane reveals a pattern with approximately 6-fold rotational symmetry for the α,δ-SCPN (Figure 7A). Those peaks correspond with the region of the C_α_ of the α-amino acids, probably because of the proximity of the amino and carboxyl groups. The presence of the two methylene groups of the cyclohexyl moieties results in a dry region next to them, where the water density is practically zero. Additionally, water seems to be more likely situated near the backbone than in the center of the pore, probably induced again by the influence of the polar character of the amino and carboxyl groups. For the α,γ-SCPN the water density shows an approximately 2-fold rotational symmetry, with three columns following a pattern wet–dry–wet (Figures 7A and 8SIA). This anisotropic profile is surprising given that the inside of this hollow structure has a 4-fold rotational symmetry. Interestingly, the origin of this asymmetry in the inner water density seems to come from the presence of Gln in the sequence, which induces an asymmetric outer surface of the channel. The replacement of the Gln by a Trp reveals a much more symmetric profile (Figure 9SI), confirming this hypothesis and highlighting that the exterior of the channel can influence the internal water arrangement. A similar effect is also observed for the α,δ-SCPNs, because the densities are not exactly 6-fold symmetric either; however, it is not so marked, probably because of the larger number of amino acids composing the sequence, which result in a larger diameter. A related phenomenon has been previously observed in a CNT - the flow is increased by the insertion of a point charge just outside the nanotube.^82^

The water profile along the *z*-coordinate reveals a higher density in the region between the CP planes for both nanotubes (Figures 7B and 8SIB), which could be explained by the larger radius found in this region (Figure 4SIB). It is important to note that the same density pattern is reproducible and present regardless of the water model. However, the densities are slightly greater following the sequence TIP3P < TIP4P < TIP4P/2005 ≈ OPC, probably because of the differences in water diffusion mentioned above.

### Ion Transport Analysis

As mentioned above, one of the most important applications of these systems is their insertion into a lipid bilayer in order to act as transmembrane ion channels. As has been found with similar nanotubes, there is a strong selectivity for cations in the α,γ-SCPNs, attributed to the negatively charged carbonyl oxygens inside this type of channel (Figures 8A, 10SIA, and 11SIA).^71,72^ For the α,δ-SCPNs this cation selectivity is less pronounced, as indicated by the presence of both cations (Na^+^) and anions (Cl^–^) in the nanotube region (Figures 8B, 10SIB, and 11SIB). This may be due to the increase in nanotube diameter as well as the greater exposure of hydrophobic groups to the lumen of the pore, because it has been suggested that hydrophobic contacts may favor chloride over cations.^30,83^ However, the number of cations inside remains considerably higher than that of anions. It is also noticeable that most of the anions entering α,δ-SCPNs are paired with the corresponding cation, as indicated by the *z*-coordinates of those ions from Figures 10SI and 11SI. Globally, there is also a difference in the number of cations inside each of the nanotubes, being higher for those containing δ-residues, in agreement with their larger diameter. Focusing on water models, in the simulations with TIP3P the number of different cations entering the nanotube is significantly higher, which could suggest a correlation between a faster diffusion and a higher number of ions entering the nanotube (Figure 12SI). No large differences have been found among the remaining models, with the number of cations always being smaller than those found for the TIP3P model.

Finally, the coordination of the cations inside the nanotube was evaluated by analyzing the number of oxygens within their first coordination layer (Figure 13SI). This shows that for both nanotubes a number of water molecules are coordinated to the cations when they are inside the SCPN, with no differences between the different water models. Additionally, although the same coordination number is observed for both peptides (6), the oxygens come from different molecules depending on the type of SCPN. For the α,δ-SCPN, it has been found that the six interactions correspond to water molecules. However, in the case of the α,γ-SCPN, the cations are coordinated to five water molecules and one carbonyl group, probably because of the smaller radius of this SCPN.

## Conclusions

A systematic molecular dynamics simulation study comparing the behavior of four water models (TIP3P, TIP4P, TIP4P/2005, and OPC) inside two sizes of self-assembled cyclic peptide nanotubes (α,γ-SCPN and α,δ-SCPNs) has been carried out. This has enabled us to investigate how the water model affects the simulated behavior of supramolecular tubes acting as transmembrane channels. All the SCPNs preserve their tubular structure across all the trajectories, independent of the water model selected. However, the results show that the dynamics of the water molecules and their interactions with the cyclic peptides present in the nanopores depend on the water model employed.

TIP3P exhibits the fastest dynamics, as indicated by the higher mean displacement values and significantly shorter nanotube filling times, followed by TIP4P. Interestingly, the two more modern water models OPC and TIP4P/2005, which both provide good results in bulk water, show quite similar dynamic properties, as can be concluded from the analysis of the velocities of the encapsulated waters, from the survival probability inside the nanotube, and from the H-bond lifetimes with the SCPN. The velocity of the water molecules confined inside the channel was TIP3P > TIP4P > TIP4P/2005 ≈ OPC, independent of the nanotube model. This trend is in accordance with that for bulk water, indicating that the confined environment studied here did not have a significant impact on this overall trend.

The density of the water inside the channels is quite similar for all the water models. Interestingly, we have found that the exterior of SCPNs can influence the inner water arrangement and thus the internal behavior of the transmembrane channel. This finding may allow the modulation of channel permeability by modifying its external surface when inserted into a lipid bilayer.

Furthermore, both nanotubes exhibit a selectivity for cations over anions, although this is more pronounced for α,γ-SCPNs as these completely block the passage of chloride ions. In the case of α,δ-SCPNs, some anions enter the channel, probably because of its higher internal radii and the greater exposure of hydrophobic residues toward the inner cavity. Additionally, the choice of water model also affects the number of ions found inside the channels. The simulations with TIP3P exhibit a higher number of cations entering the nanotube, highlighting the role of the water model in ion transport properties. Additionally, it has been shown that the cations inside the channel are coordinated to water molecules (6 in the case of α,δ-SCPNs, 5 for α,γ-SCPNs), with no differences between the water models. For the narrower nanotube, the vacant coordination position is occupied by an oxygen from the carbonyl groups of the skeleton of the peptide, suggesting that the pore size of such a SCPN is too narrow to transport Na^+^ while keeping its complete first hydration layer intact.

The significance of this study resides in the importance of research into transmembrane ion channels formed by cyclic peptides. Our results show that ion and water transport rates depend on the water model employed for the simulations. The obtained results follow the expected trend for bulk properties. However, it is important to note that none of these models was specifically designed for simulations of water in nanotubes, and in the absence of wet-lab experimental structural and dynamic data of water in SCPNs, it is not possible to assess which of them more accurately models reality. We therefore propose that this data set could be used as a point of reference for wet-lab experiments. Such a comparison is important for the validation of water models in confined systems.

## Methods

The starting geometries of the employed cyclic peptides were taken from previous works.^74^ Both SCPNs are composed of eight units of stacked CPs. The antiparallel and parallel configurations were used for α,γ-SCPN and α,δ-SCPN, respectively, with the decision being based on preliminary studies.^74^ For the standard amino acids and the ions, the parameters from the AMBER99SB-ILDN force field were used.^84^ For the atoms of the nonstandard amino acids (δ and γ residues), RESP/6-31G(d) charges were derived, as in the development of the original AMBER force fields. The van der Waals parameters were obtained from the GAFF force field using standard Lorenz–Bertelot combination rules.^85^ For the POPC lipids, the parameters derived by Joakim Jämbeck (Lipidbook) were used.^86−88^ In all simulations, the water molecules in the hydrophobic region of the lipids, together with the waters of the inner cavity of the nanotube, were removed prior to the start of the simulation. In the first step of the simulation, the SCPN lumen was therefore completely dry. A solution of 0.15 M NaCl was added in all cases. The initial size of the simulation box for all cases was 12.7 × 13.4 × 8.5 nm^3^ and contained 131 Na^+^, 131 Cl^–^, and ∼24 000 water molecules. The number of lipids differed depending on the SCPNs used: for the α,δ-SCPNs, the simulation box contained 407 lipids, whereas for the α,γ-SCPNs, it contained 479 lipids. This variation arises from the difference in the diameter of the SCPNs.

All simulations were performed with the GROMACS 2018.3 package.^89^ All systems were first minimized, followed by an unrestrained production run of 50 ns, with a time step of 2 fs. No restraints were applied to the peptides at any step, allowing the free movement of all atoms. Five replicas, starting from the same coordinates but differing in the initial velocities, which were randomly assigned from a Boltzmann distribution in the first step of the production run, were made for each system, bringing the total number of simulations to 40 : 20 for each nanotube (α,γ-SCPN and α,δ-SCPN), with 5 for each water model (TIP3P, TIP4P, TIP4P/2005, and OPC). An *NPT* ensemble (constant pressure and temperature) was employed at 1 bar pressure, using the semi-isotropic Parrinello–Rahman barostat, and a temperature of 300 K, using a V-rescale thermostat (*i.e*., temperature coupling using velocity rescaling with a stochastic term).^90,91^ In all simulations, all bond vibrations were removed employing the LINCS algorithm.^92^ For treating the long-range electrostatics, the Particle Mesh Ewald method (PME) was used, using a direct-space cutoff of 1.0 nm and a grid spacing of 0.12 nm.^93^ The van der Waals interactions were calculated using a cutoff of 1.0 nm.

All initial coordinates, topologies and *mdp* files needed for reproducing these simulations are available in *Zenodo* through the following link: 10.5281/zenodo.4420015 (DOI: 10.5281/zenodo.4420015)

The data obtained in the simulations were analyzed using GROMACS tools and locally written code using the Python MDAnalysis library.^94,95^ The molecular graphic pictures of the systems were prepared using the molecular visualizer VMD and PyMOL, and the pore size was calculated using the package CHAP.^96−98^

The RMSD calculations were carried out using the GROMACS tool. The initial frame of the trajectory was taken as the reference point, as in this case the tubular shape was expected to be perfectly formed. In order to avoid fluctuations provoked by the side chains, only the backbone was considered.

The tilt angle was defined as the angle between the normal of the membrane and a vector which links the center of the CPs to the edges of the nanotube. For calculation of the mean displacements, 160 windows of 100 ps were defined starting at different points of the simulations. For each window, the total amount of movement of each water molecule was calculated, averaging over all these molecules. Only the waters which were inside the nanotube during the whole window were considered. The probability distributions (*P*) presented for these two magnitudes were calculated using the python library Numpy.^99^

The survival probability of the waters inside the nanotube represents the likelihood of one water molecule remaining encapsulated after a certain time, following the approach described by Liu *et al.*(100) The autocorrelation function for the H-bond lifetime has been obtained using the description proposed by Rapaport, being here presented as the intermittent H-bonds.^101^ These two calculations are available in the *Water Dynamics Analysis* section of MDAnalysis.^102^

For the water density maps, a grid in the *xy* or *xz* planes was defined, counting the number of times that a water molecule was presented in each position. In order to obtain a proper comparison, the results were normalized by taking the most populated point of all simulations as a reference point.

The *z*-coordinate representation of the ions, as well as the total number of cations which enter the nanotube and the number of molecules coordinated to them, were obtained using local Python code. The coordination numbers of the cations inside the SCPN were calculated by counting the number of oxygens at a distance smaller than a defined cutoff, corresponding to the first coordination layer of Na^+^. This value was obtained from the RDF calculation of oxygen of the waters taking as reference all the cations (including those outside the nanotube), using the GROMACS tool (Figure SI14).

## Supporting Information Available

Additional analyses of simulations (RMSD, inner radius, *z*-coordinate for anions, *etc*.) (PDF)

## Notes

All initial coordinates, topologies, and *mdp* files needed for reproducing these simulations are available in *Zenodo* through the following link: 10.5281/zenodo.4420015 (DOI: 10.5281/zenodo.4420015).
