# Supplementary material for: Effect of Water Models on Transmembrane Self-Assembled Cyclic Peptide Nanotubes
Source: ACS Nano. 2021 Mar 19;15(4):7053–64. doi: 10.1021/acsnano.1c00155 (PMC8485350; doi:10.1021/acsnano.1c00155)
Supplement: Supplementary file 1 — nn1c00155_si_001.pdf [file nn1c00155_si_001.pdf]

SUPPORTING INFORMATION FOR:

Effect of Water Models on Transmembrane Self-Assembled Cyclic Peptide Nanotubes

*Martin Calvelo,[A] Charlotte I. Lynch,[B] Juan R. Granja[A] Mark S. P. Sansom,[B] and Rebeca Garcia-Fandiño.[A]**

[A]Center for Research in Biological Chemistry and Molecular Materials (CIQUS), University of Santiago de Compostela, 15782 Santiago de Compostela, Spain

[B]Department of Biochemistry, University of Oxford, South Parks Road, Oxford OX1 3QU, United Kingdom

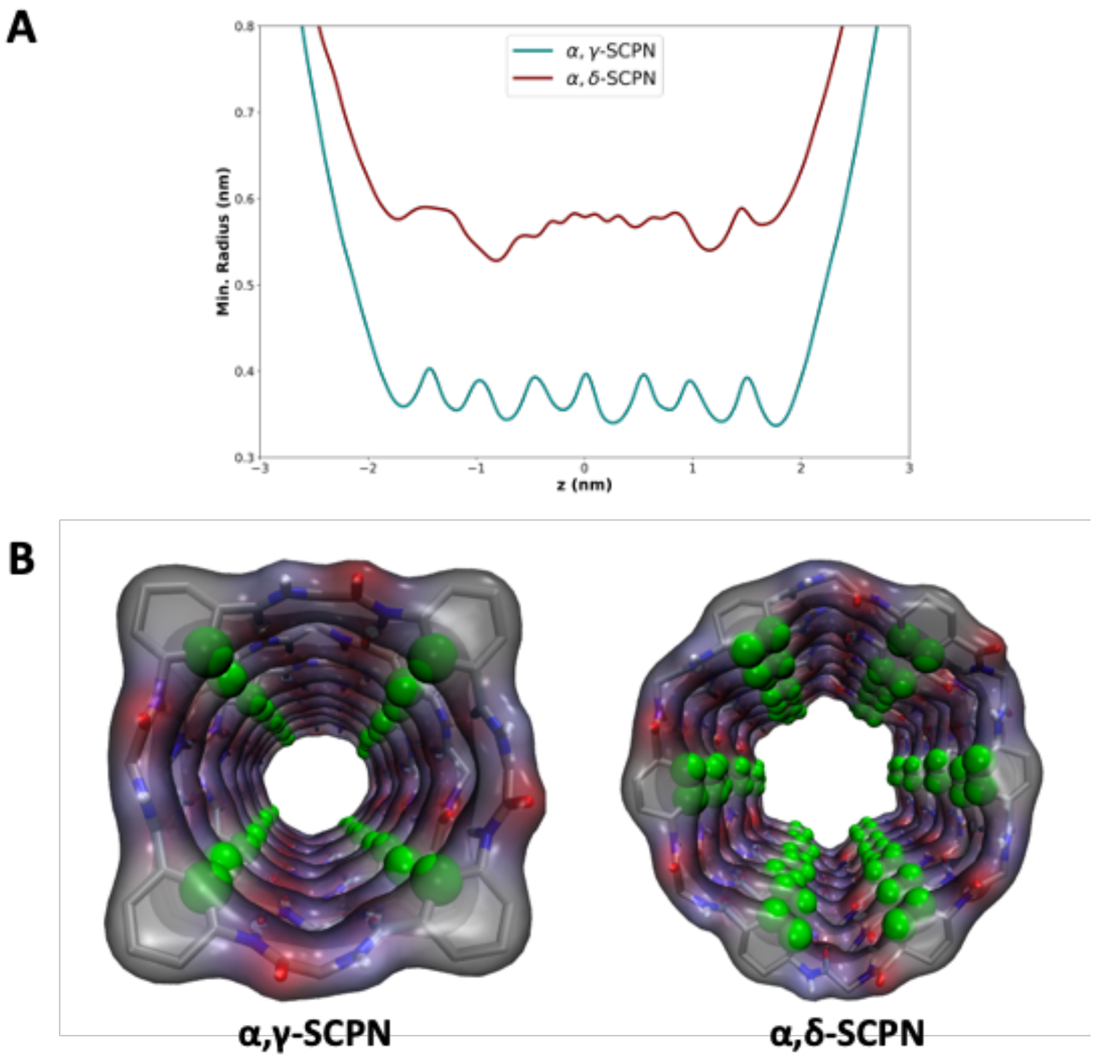

Figure 1SI. A) Minimum inner radius of the α,γ-SCPN and α,δ-SCPN at the beginning of the simulation (*time = 0*). **B)** Detail of the inner cavity of both SCPNs, highlighting the hydrophobic moieties oriented inwards in green.

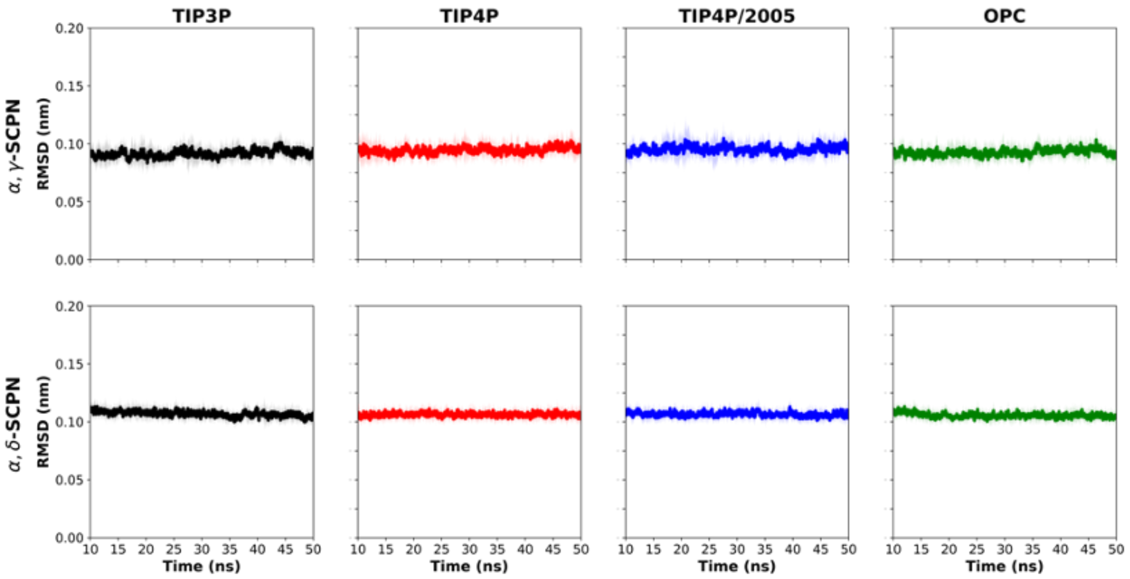

Figure 2SI. Average backbone RMSD among the five replicas of both SCPNs in TIP3P, TIP4P, TIP4P/2005 and OPC, respectively. Each water model is displayed with a different colour. Standard deviations are shown in a paler colour.

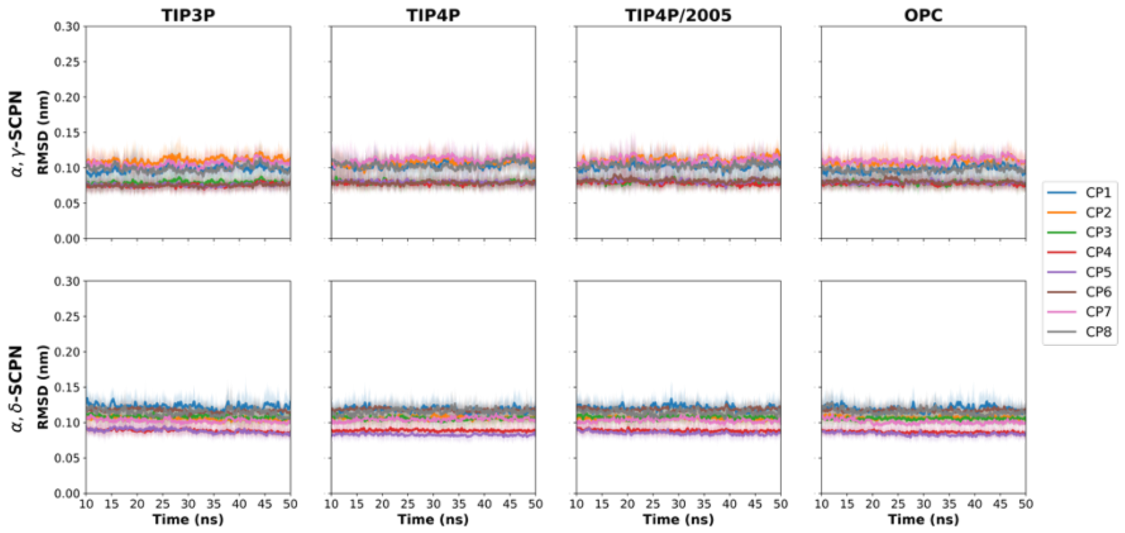

Figure 3SI. Average backbone RMSD among the five replicas of both SCPNs split by CP in TIP3P, TIP4P, TIP4P/2005 and OPC. Standard deviations are shown in a paler colour.

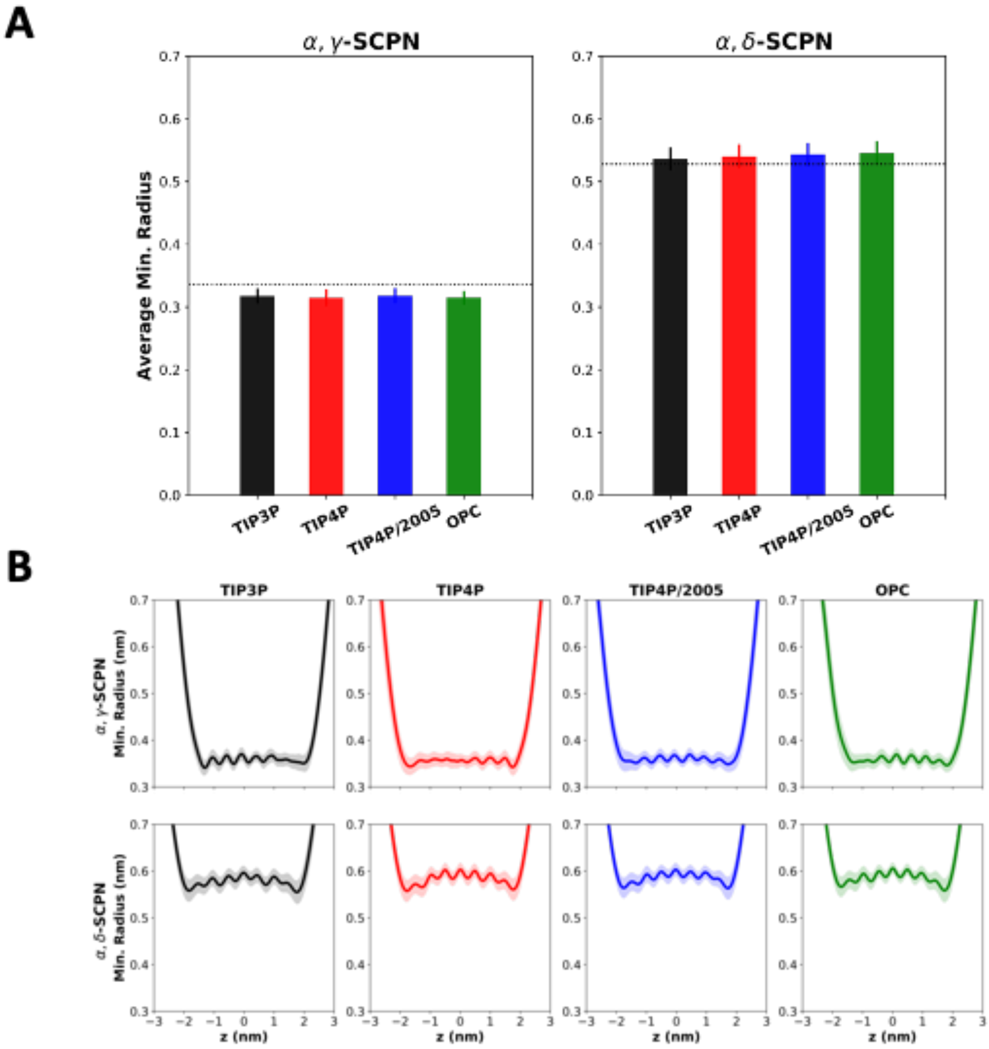

Figure 4SI. Minimum inner radius averaged over the five replicas, as well as the standard deviation, for both SCPNs in TIP3P, TIP4P, TIP4P/2005 and OPC presented as **A)** a histogram and **B)** along the z axis of the nanotube.

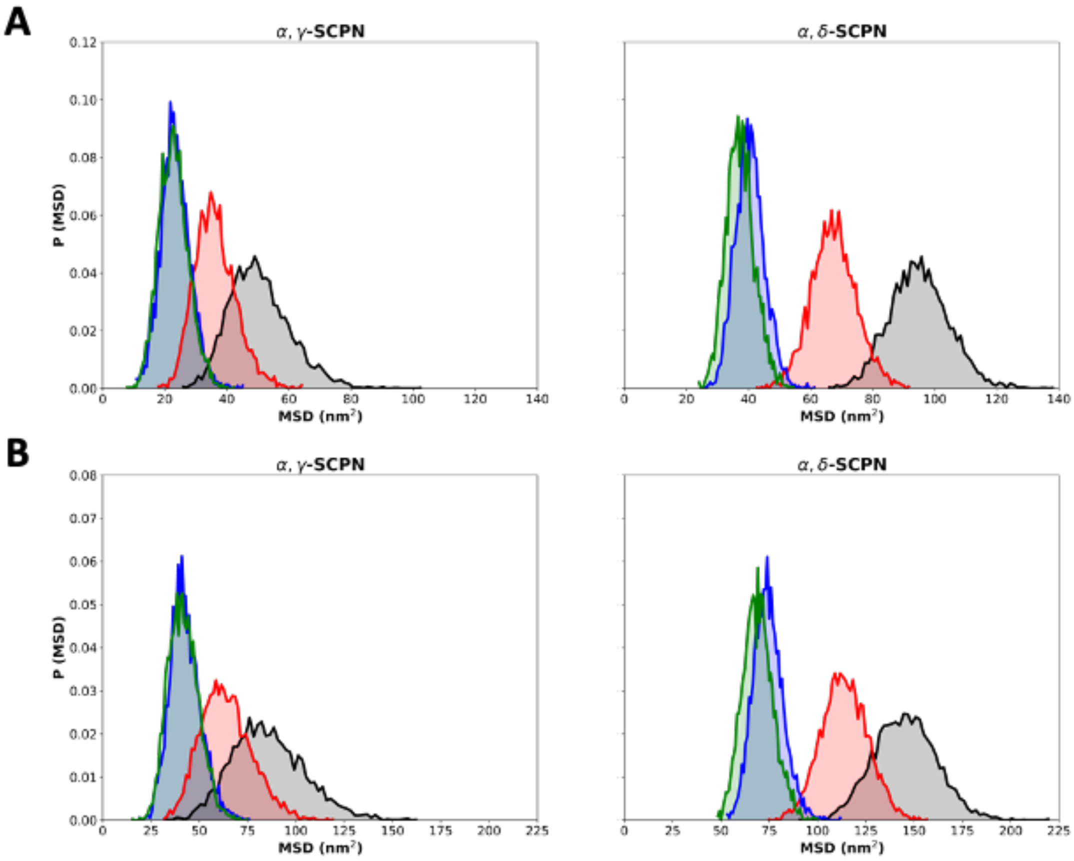

Figure 5SI. Probability distribution of the MSD of waters inside α,γ-SCPNs and α,δ-SCPNs during windows of 200 ps (**A**) and 500 ps (**B**) along the last 40 ns of all replicas for the different water models studied. Each water model is displayed with a different colour (TIP3P in black, TIP4P in red, TIP4P/2005 in blue and OPC green).

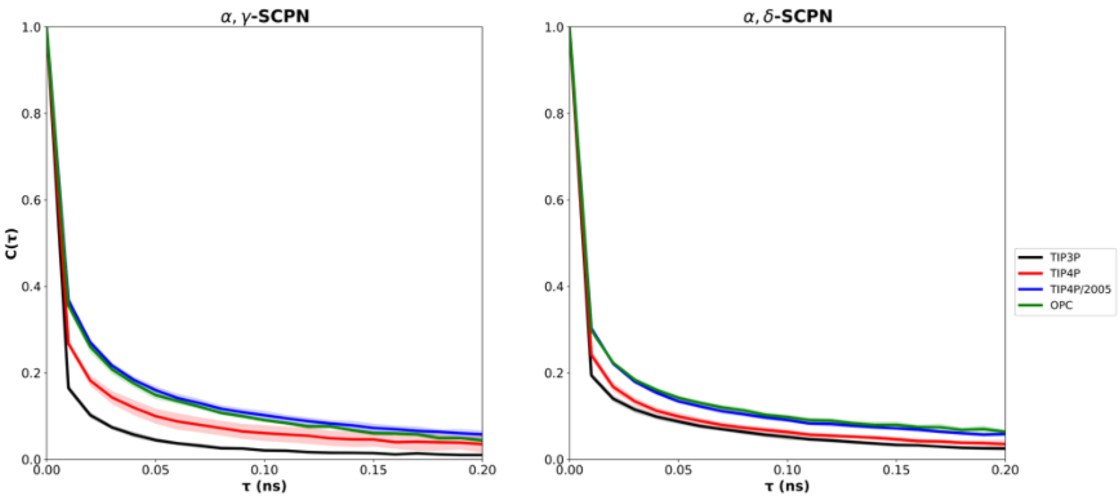

Figure 6SI. H-bond lifetime between both SCPNs and water molecules in TIP3P, TIP4P, TIP4P/2005 and OPC, averaged over the five replicas. Each water model is displayed in a different colour (TIP3P in black, TIP4P in red, TIP4P/2005 in blue and OPC green). Standard deviations are shown in a paler colour.

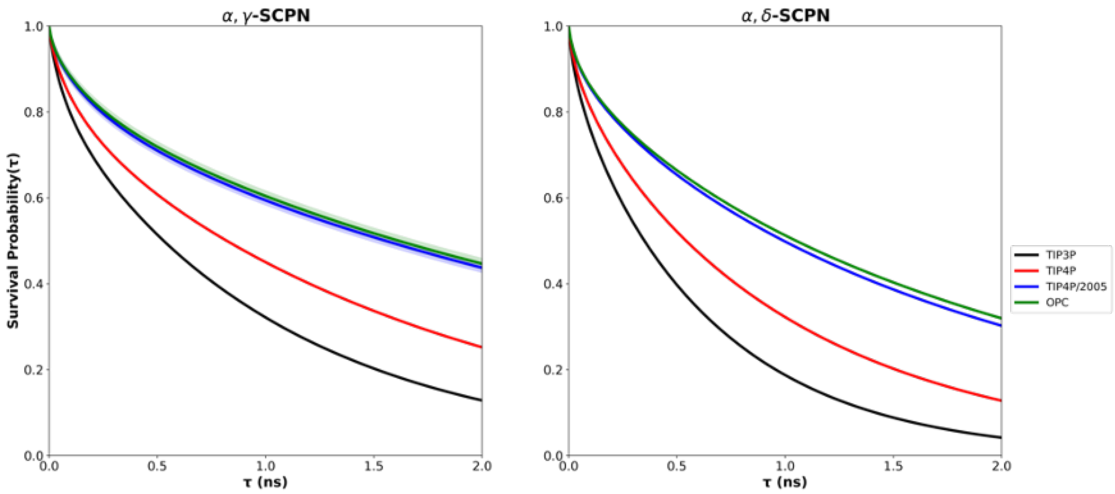

Figure 7SI. Survival probability of water molecules inside both SCPNs in TIP3P, TIP4P, TIP4P/2005 and OPC, averaged over the five replicas. Each water model is displayed in a different colour (TIP3P in black, TIP4P in red, TIP4P/2005 in blue and OPC green). Standard deviations are shown in a paler colour.

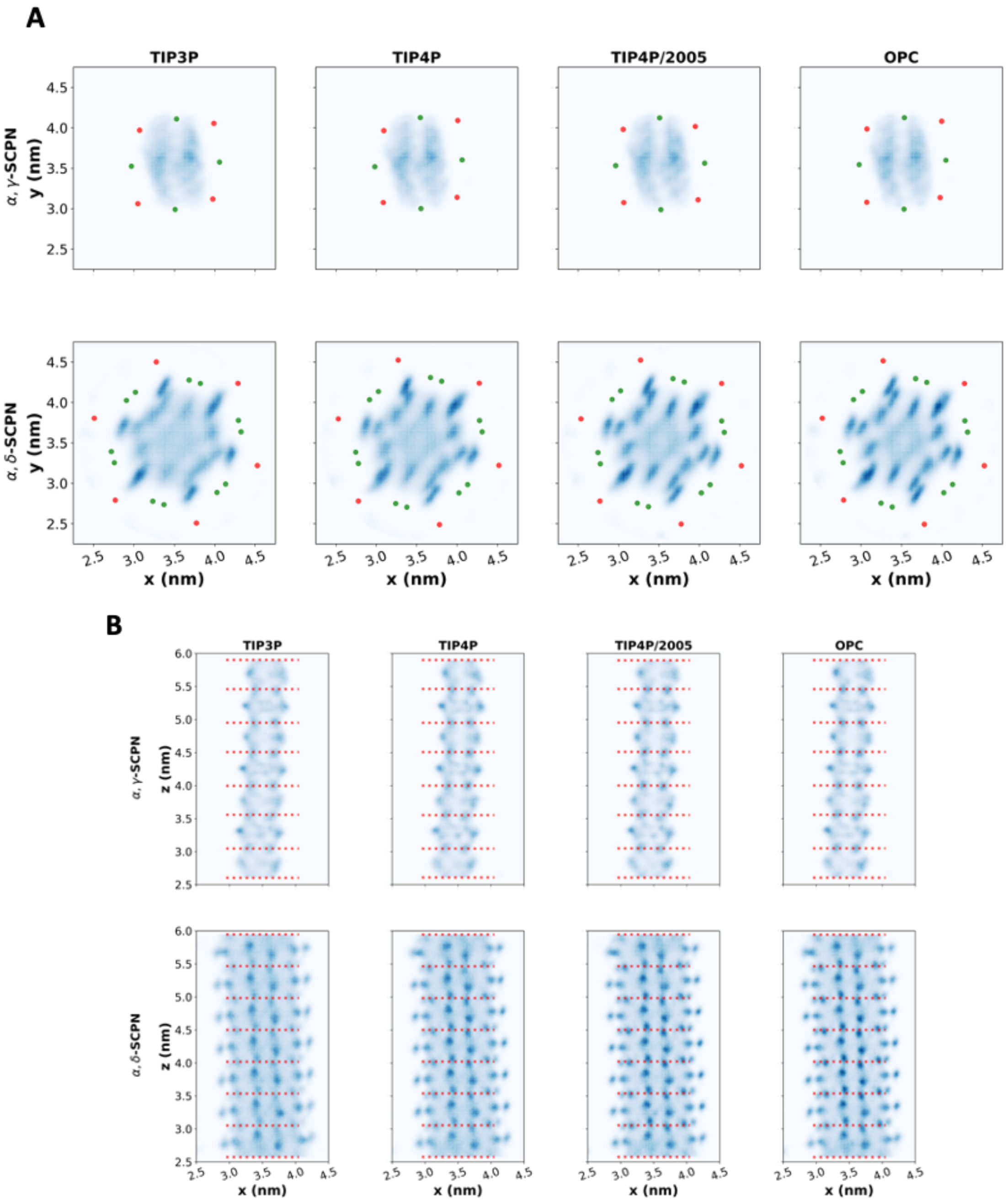

Figure 8SI. A) XY-Density profile of the water molecules inside both nanotubes. The averaged positions of the C_α of the α-amino acids and the C of the inwards methylenes of the non-natural residues are highlighted in red and green, respectively. **B)** ZX-Density profile of the water molecules inside both nanotubes. The averaged Z-coordinates of the α-carbons of each CP are highlighted with a red dashed line.

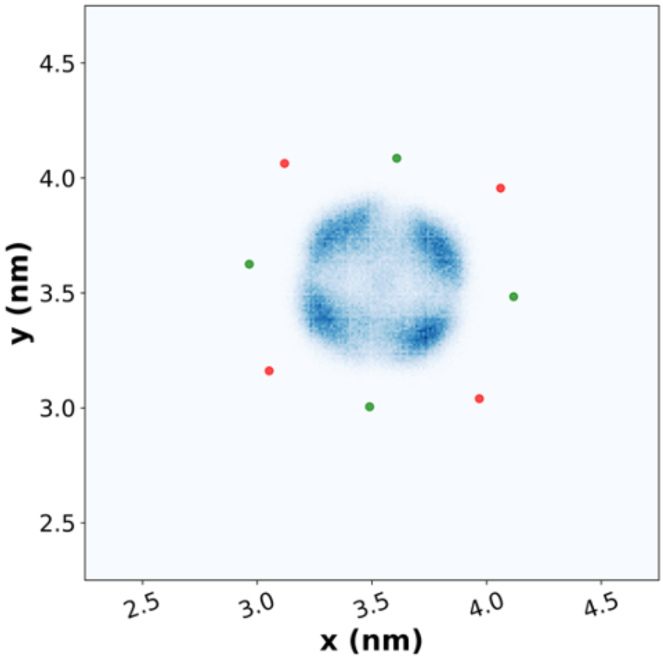

Figure 9SI. XY-Density profile of the water molecules inside a α,γ-SCPN in which all the α-amino acids are Trp, using the TIP3P model. The averaged positions C_α of the α-amino acids and the C of the inwards methylene groups of the non-natural residues are highlighted in red and green, respectively.

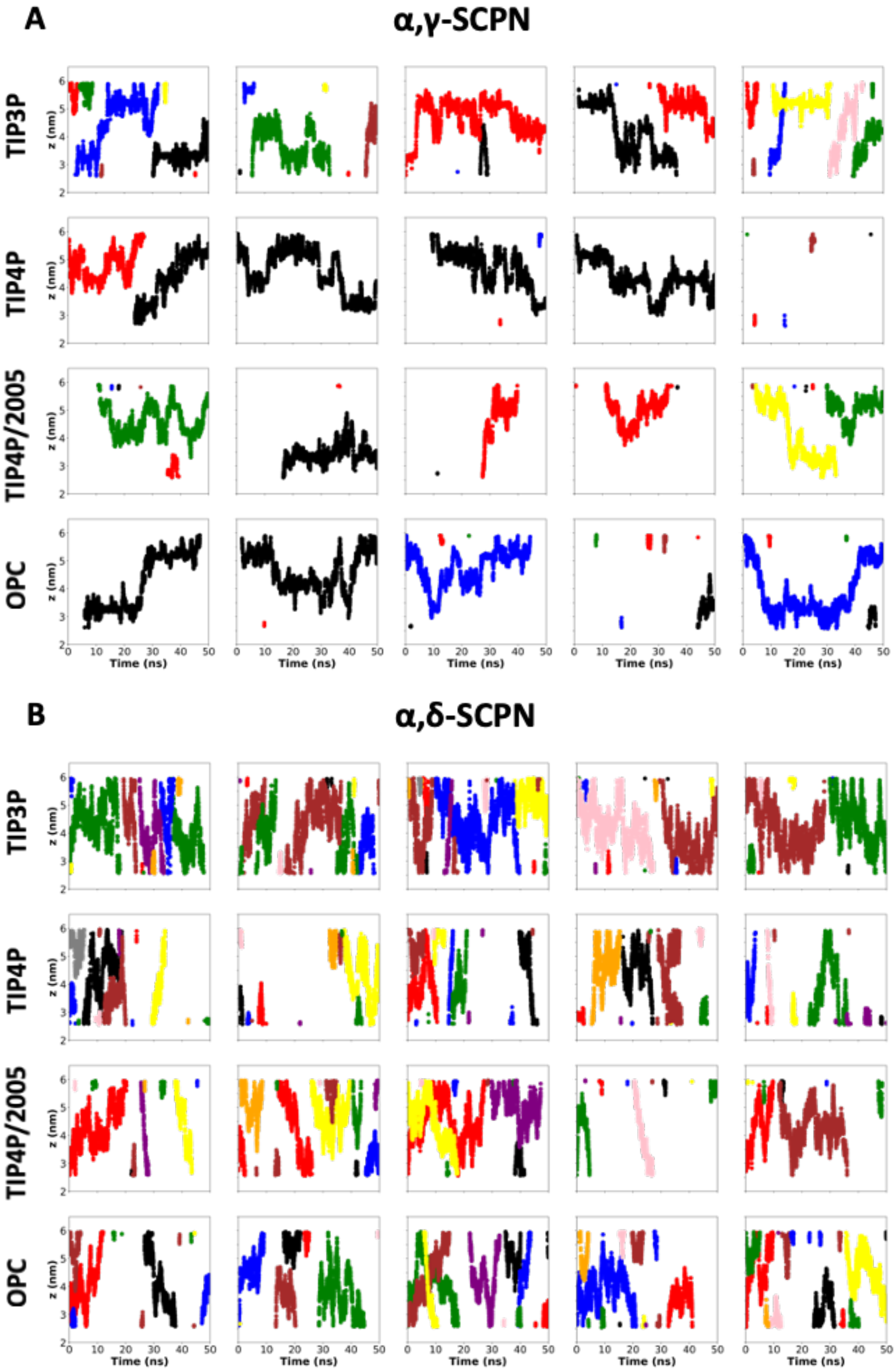

Figure 10SI. Z-coordinate for each of the cations inside the α,γ-SCPN (**A**) and α,δ-SCPN (**B**) along the 50 ns trajectory. The nanotube z-region is taken to be between ≈2–6 nm. Each column corresponds to a different replica. Each colour corresponds to a different ion.

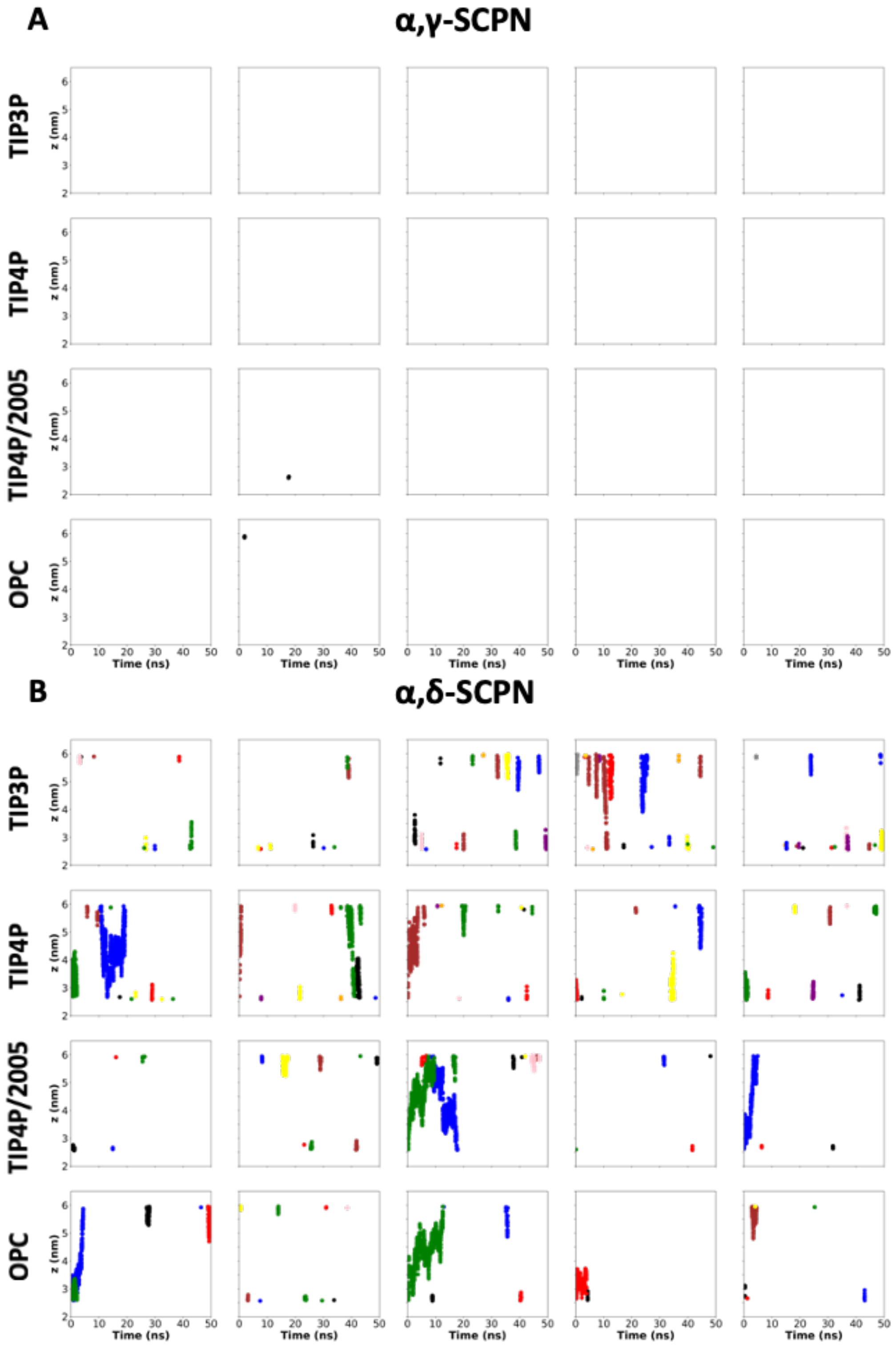

Figure 11SI. Z-coordinate for each of the anions inside the α,γ-SCPN (**A**) and α,δ-SCPN (**B**) along the 50 ns trajectory. The nanotube z-region is taken to be between ≈2–6 nm. Each column corresponds to a different replica. Each colour corresponds to a different ion.

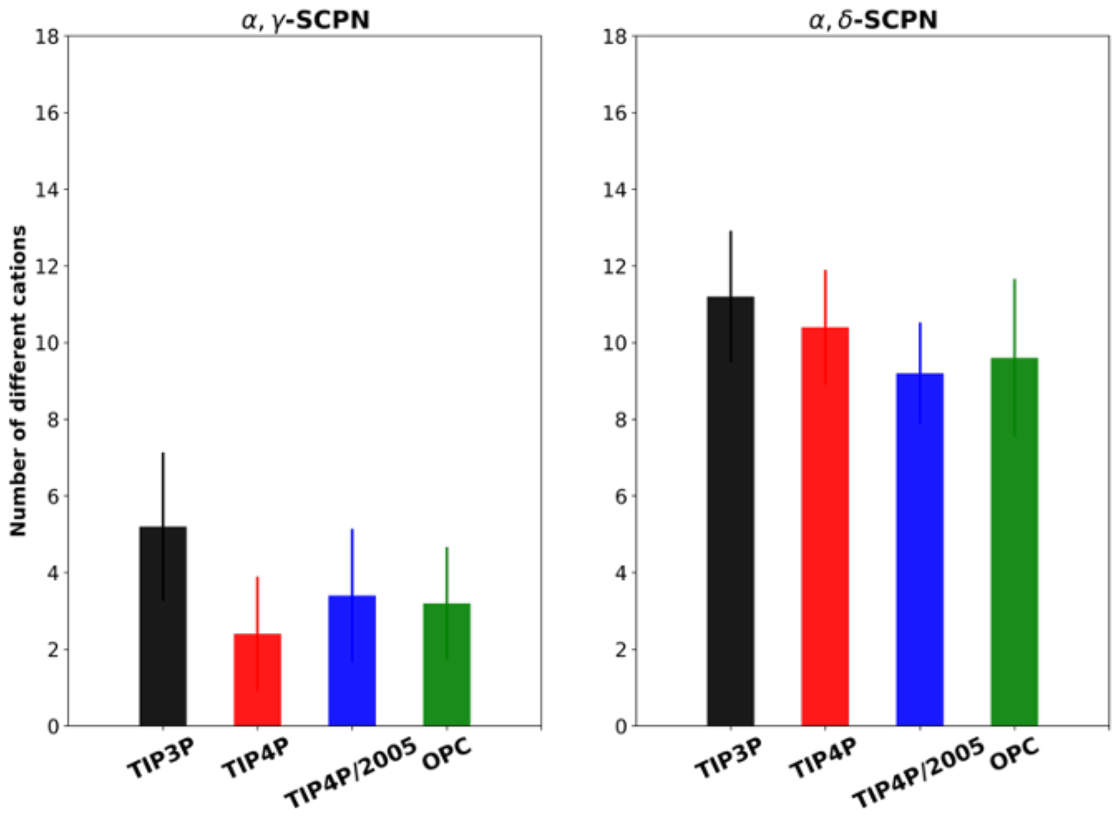

Figure 12SI. Total number of different cations which enter both nanotubes along the 50 ns of simulation averaged over the five replicas in TIP3P, TIP4P, TIP4P/2005 and OPC. Standard deviations are given as error bars.

A

	α,γ-SCPN		α,δ-SCPN	
	Water	C=O (BB)	Water	C=O (BB)
TIP3P	5±1	1±1	6±1	0
TIP4P	5±1	1±1	6±1	0
TIP4P/2005	5±1	1±1	6±1	0
OPC	5±1	1±1	6±1	0

B

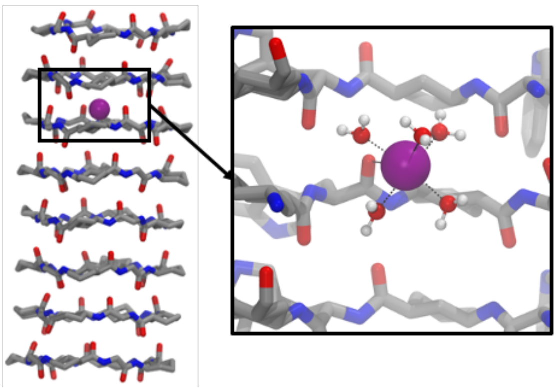

C

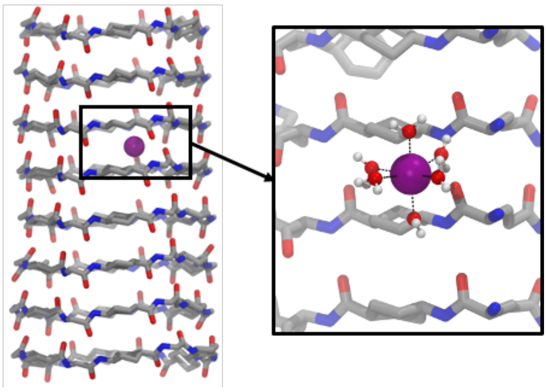

Figure 13SI. A) Averaged number of oxygens from the water and the carbonyl groups of the amino acids of the backbone of the SCPNs coordinated to the Na^+ cations inside the nanotube. This number was defined as the number of oxygen atoms within a cut-off of 3.2 Å (approx. the end of the first coordination sphere observed in the RDF calculation, see below). **B)** Detail of the coordination pattern of a Na^+ cation inside the α,γ-SCPN, in which 5 waters and 1 carbonyl group are coordinated to a cation (purple sphere). **C)** Detail of the coordination pattern of a Na^+ cation inside the α,δ-SCPN, in which 6 waters are coordinated to a cation (purple sphere).

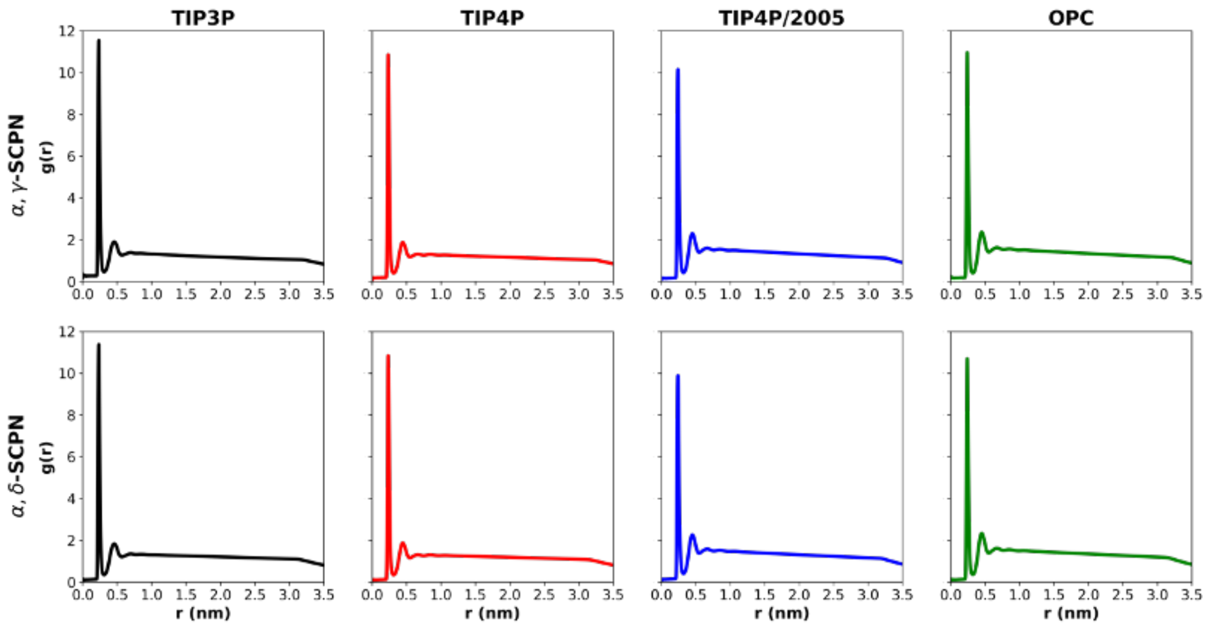

Figure 14SI. Radial distribution function (RDF) of the oxygen of the water molecules around the Na^+ cations calculated for the 50 ns of simulation and averaged over the five replicas in TIP3P, TIP4P, TIP4P/2005 and OPC.
